# Early Horizontal Deceleration Ability Across Multiple Steps and Approach Speeds in Multidirectional Team Sport Athletes

**DOI:** 10.1002/ejsc.70224

**Published:** 2026-07-28

**Authors:** Luke Hitchens, Jasper Verheul

**Affiliations:** ^1^ Cardiff School of Sport and Health Sciences Cardiff Metropolitan University Cardiff UK

**Keywords:** athletic performance, braking, external kinetics, joint kinematics, targeted training

## Abstract

Horizontal deceleration ability (HDA) is a critical component of multidirectional team sport performance. This study aimed to identify the interaction between external kinetic and kinematic determinants of HDA across multiple deceleration steps and approach speeds. Nineteen multidirectional team sport athletes completed five maximal horizontal decelerations at 100%, 85%, and 70% of maximal sprint velocity, while three‐dimensional ground reaction forces (GRF) and kinematics were captured during the first three deceleration steps. HDA was quantified as the average rate of velocity reduction (i.e., deceleration) within each step and cumulatively across steps 1–3 (early HDA). Greater within‐step deceleration significantly correlated with greater mean horizontal GRF (HGRF) and horizontal‐to‐vertical (H‐V) force ratio across all steps and approach speeds (*r* = 0.56–0.95, *p* = 0.001–0.013). Mean HGRF in step 1 (*β* = 0.58–0.83, *p* = 0.01) and H‐V force ratio in step 3 (*β* = 0.50–0.68, *p* = 0.01) were the strongest predictors of early HDA. Greater peak hip and knee flexion angles in step 1, and a lower centre‐of‐mass height, increased anterior foot placement and a more negative shank inclination at touchdown in steps 2 and 3 were associated with greater mean HGRF and H‐V force ratio, with stronger associations observed at slower speeds (*r* = 0.49–0.90, *p* = 0.001–0.033). These findings indicate that early HDA is underpinned by step‐specific kinetic and kinematic strategies. Practitioners should target the attenuation of horizontal force magnitude in step 1 via greater peak hip and knee flexion and enhance horizontal force orientation in later steps by optimising touchdown postures, such as centre‐of‐mass height, anterior foot placement and shank inclination.

## Highlights

- Athletes can adopt more effective braking strategies once momentum has been reduced, as the relationship between kinematic and kinetic determinants of horizontal deceleration progressively strengthened during later deceleration steps and at slower approach speeds.
- Step‐specific assessment and training of deceleration mechanics are essential, as distinct kinetic and kinematic deceleration strategies were observed across successive deceleration steps.
- Coaching strategies that encourage athletes to increase peak hip and knee flexion during the initial step of braking can enhance deceleration performance, since this first step of deceleration requires the greatest attenuation of horizontal force magnitude.
- A focus on optimised body posture at touchdown, including a reduced centre‐of‐mass height, a more anterior foot placement and greater negative shank inclination, is most beneficial during subsequent deceleration steps, as it promotes a more favourable force vector orientation—a key kinetic determinant of deceleration performance.

## 1 Introduction

Horizontal deceleration ability (HDA), defined as an athlete's capacity to rapidly reduce whole‐body momentum, is a fundamental locomotor skill in multidirectional team sports (Harper et al. 2019; Harper et al. 2022). High‐intensity horizontal decelerations (> 2.5 m·s^−2^), especially, account for a large proportion of the total running actions in multidirectional team sports and are more frequently performed than equivalent‐intensity accelerations (Akenhead et al. 2013; Dalen et al. 2016; Oliva‐Lozano et al. 2020; Vigh‐Larsen et al. 2018). Athletes who can effectively decelerate are better equipped to respond to an opponent's actions, stop to execute sport‐specific technical skills, or change running direction (Ade et al. 2016; Harper et al. 2022; Harper et al. 2019). Accordingly, horizontal decelerations have been shown to be one of the most important movements preceding goals and a higher frequency of decelerations is positively associated with match outcome in professional football (soccer) (Martínez‐Hernández et al. 2023; Rhodes et al. 2021). Given the critical importance of horizontal deceleration manoeuvres for multidirectional‐sport performance, there is a pressing need to identify the specific kinetic and kinematic strategies that underpin effective braking. Doing so will enable practitioners to design targeted training interventions for optimising movement mechanics and enhancing performance.

To rapidly reduce whole‐body momentum, horizontal decelerations require athletes to generate and attenuate considerably greater ground reaction forces (GRF) than any other locomotor movement (e.g., change of direction, acceleration and high‐speed running; Lozano‐Berges et al. 2021; Verheul et al. 2019, 2021). Both the magnitude of horizontal GRF and the orientation of the force vector have been identified as key determinants of deceleration performance (Li et al. 2025; Harper et al. 2022). According to a conceptual framework proposed by Harper et al. (2022), these kinetic determinants are influenced by technical movement strategies, whereby rapid postural adjustments, including lowering the centre of mass and increasing anterior foot placement, facilitate the production and orientation of braking forces. Understanding this interaction between kinetic and kinematic biomechanical determinants of HDA is thus essential for informing deceleration‐specific training interventions.

Horizontal force production during the initial braking steps appears especially influential, as early deceleration ability (i.e., performance within the first 50% of the deceleration sequence) strongly predicts overall deceleration ability (Norman et al. 2025). This emphasises the need to examine how athletes produce and orient horizontal forces during the early deceleration steps, specifically the first three steps, which represent ∼50% of the sequence in high‐approach‐speed decelerations (Falch et al. 2020). However, most biomechanical research to date has focused on a single deceleration step (Cesar and Sigward 2016; Cross et al. 2024; Dix et al. 2020; Jordan et al. 2021; Lozano‐Berges et al. 2021; Verheul et al. 2024), with only one recent study investigating multiple early deceleration steps (Li et al. 2025). While this recent study provided important insights into step‐specific external kinetics, it did not include any analysis of whole‐body or joint‐level kinematics. Consequently, it remains unclear which movement strategies underpin the step‐specific kinetic determinants of HDA. This is an important limitation, as rapid postural adjustments influence horizontal force application and deceleration ability (Cesar and Sigward 2016; Dos' Santos et al. 2020; Harper et al. 2022) and are potentially modifiable through training (Morin et al. 2011). Furthermore, as whole‐body momentum progressively decreases with each successive step, the mechanical demands, and therefore the determinants of effective braking, are also likely to vary throughout the deceleration sequence. Therefore, a comprehensive analysis of kinetic and kinematic variables across the early deceleration steps is warranted to inform the design of targeted, step‐specific training interventions aimed at enhancing HDA.

During multidirectional team sports, horizontal decelerations are performed under a range of tactical and environmental constraints, resulting in braking manoeuvres being initiated from a wide spectrum of approach velocities (Hewit et al. 2011). Given that the majority of match play is performed at submaximal running intensities rather than sprinting, many decelerations are likely to be initiated from submaximal velocities (Bloomfield et al. 2007). Different approach speeds may impose distinct mechanical demands and are underpinned by different biomechanical determinants of performance. While several studies have compared decelerations from varying approach speeds, these studies have primarily focused on kinematic characteristics of deceleration (Chen et al. 2024; Jordan et al. 2021; Norman et al. 2025), leaving the interaction between kinematic and kinetic determinants of HDA relatively underexplored. Notably, Norman et al. (2025) recently observed that some athletes excelled at decelerating from certain approach speeds but not others, highlighting that deceleration ability is likely to be underpinned by different kinetic and kinematic strategies at different approach speeds. Therefore, to support the development of effective performance‐enhancing training interventions, it is necessary to identify how the biomechanical demands and performance determinants of HDA differ between faster and slower approach speeds.

Concurrent examinations of the external kinetics and kinematics of horizontal deceleration, across multiple steps and approach speeds, are essential to understand what constitutes effective HDA and better inform deceleration specific training. Therefore, this study aims to (1) identify the step‐ and speed‐specific external kinetic determinants and underpinning kinematic strategies of HDA, and to (2) compare kinetic and kinematic characteristics across early deceleration steps and approach speeds. Given that whole‐body momentum and braking demands reduce with approach speed and across successive deceleration steps, it was hypothesised that the relationships between kinematic strategies and the kinetic determinants underpinning early HDA would become stronger during later deceleration steps and at slower approach speeds.

## 2 Methods

### 2.1 Participants

Nineteen trained athletes participated in this study (14 males and 5 females, age 20 ± 1 years, height 1.77 ± 0.07 m, body mass 80 ± 17 kg). Based on the final sample size, a post hoc power analysis (G*Power 3.1.9.7) indicated 92% power to detect a medium effect (*f* = 0.25; *α* = 0.05) for the repeated‐measures ANOVA (within factors). Both male and female athletes were included to increase the heterogeneity of the athletic cohort, although sex‐specific comparisons were not performed. Participants were all healthy, free of any lower limb injury for a minimum of 6 months and trained and competed in a multidirectional team sport (e.g., football, rugby, hockey) for 5 ± 1 hours per week. Each participant completed a readiness to exercise questionnaire and provided written informed consent prior to participation. This study was approved by Cardiff Metropolitan University Natural Ethics Committee (reference number: PGT‐9450).

### 2.2 Deceleration Protocol

**FIGURE 1:**
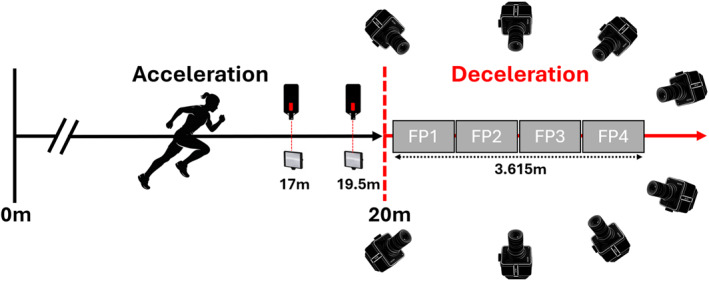
Acceleration‐to‐deceleration experimental setup used to assess early HDA. Timing gates were positioned at 17 and 19.5 m; camera icons represent vicon vue video cameras and FP denotes force platform. The acceleration and deceleration phases are indicated in black and red, respectively. Participants were instructed to initiate deceleration after crossing the 20 m line.

Following a standardised warm‐up of light running, dynamic stretching, and progressive sprints, participants completed a series of acceleration‐to‐deceleration trials. The protocol, adapted from Harper et al. (2021), required participants to accelerate 20 m from a standing start followed by a pre‐planned maximal linear deceleration initiated on a series of four ground‐embedded force platforms (Figure 1). Participants were instructed to initiate deceleration immediately after crossing the 20 m mark and to decelerate to a complete stop, holding a stationary position for 2 seconds to clearly mark the end of the deceleration phase. Additionally, to minimise force plate targeting and promote natural deceleration strategy, participants were not informed of the force plate locations and were instructed to decelerate as naturally as possible following the 20 m mark. Participants completed fifteen successful trials, five at each of three approach speeds: 100%, 85%, and 70% of their maximal 20 m sprint velocity. The submaximal speeds were calculated relative to the average peak velocity achieved across the five maximal (100%) trials. Following each trial, SmartSpeed timing gates (VALD, Brisbane, Australia) provided real‐time feedback on approach speed, which the research team used to verbally instruct athletes (i.e., ‘speed up’ or ‘slow down’) to remain within ± 5% of the target approach speed. However, the timing gate measurements were used only to provide immediate feedback during testing. Final trial inclusion was based on peak whole‐body COM velocity derived from the motion‐capture data, with only trials within ± 5% of the target approach speed included in the final analysis. In addition, only trials in which the first deceleration step was performed with the dominant limb contacting the force platforms were included. Deceleration onset was defined as the first step following a reduction in whole‐body COM velocity. Trials were separated by two‐minute rest intervals to minimise fatigue.

### 2.3 Kinematic and Kinetic Data Collection

Markerless three‐dimensional (3D) kinematic data were recorded using eight high‐resolution video cameras (Vicon Vue, Oxford, UK) sampling at 100 Hz (Figure 1). Four ground‐embedded force platforms (Type 9287BA, Kistler, Winterhur, Switzerland), topped with Mondo track surface and sampling at 1000 Hz, were used to record GRF during the early deceleration steps (i.e., first three steps). Kinematic and kinetic data were synchronously recorded with Vicon Nexus (v.2.12.1, Oxford Metrics Inc., Oxford, UK).

Raw markerless video data were processed by Theia3D (Theia Markerless Inc., Kingston, ON) where the default inverse kinematic solution was used to estimate the 3D pose. Previous studies have reported agreement between Theia3D and marker‐based motion capture of approximately 3°–7° for most lower‐limb sagittal‐plane kinematics during dynamic tasks, with inter‐session variability generally within 4° (Varcin and Boocock 2025). The Theia3D default Generalised Cross‐Validation Spline with cutoff frequency of 20 Hz was used to filter the kinematic data. The processed kinematic and synchronised GRF data were exported to c3d files for further analysis in Visual3D (v.6, C‐motion, Rockville, MD, USA), where GRF data were filtered at 50 Hz using a second‐order lowpass Butterworth filter, consistent with previous high‐intensity deceleration investigations (Verheul et al. 2024).

### 2.4 Kinematic and Kinetic Analysis

Horizontal approach velocity was initially estimated using timing gates to provide real‐time feedback during data collection; however, final trial inclusion was based on peak whole‐body COM velocity derived from the markerless 3D motion‐capture data. Trials deviating more than ± 5% from the target velocity (85% or 70%) were excluded. The first three deceleration steps were identified from vertical GRF data, with touchdown defined as the instant vertical GRF exceeded 20 N and toe‐off defined as the instant vertical GRF fell below 20 N.

**FIGURE 2:**
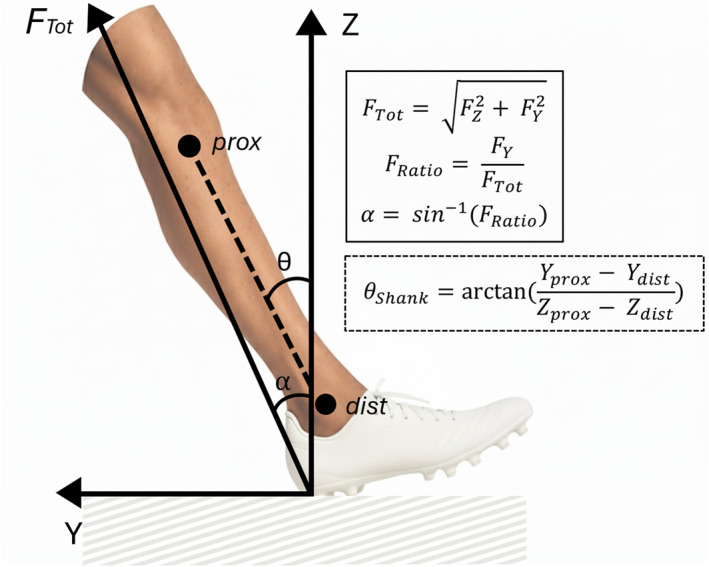
Schematic representation and mathematical expression for the H‐V force ratio ($F_{Ratio}$), angle of force inclination (*α*), and angle of shank inclination (${ϴ}_{Shank}$). Dist = distal endpoint of the tibia; Prox = proximal endpoint of the tibia.

Sagittal plane kinetic variables were derived from anteroposterior (horizontal) and vertical GRF, normalised to body weight (BW). Peak and mean forces were defined as the maximum and average forces during ground contact, respectively. Force impulses (i.e., area under the GRF curve) were calculated as the time integral of each GRF component. The mean horizontal‐to‐vertical (H‐V) force ratio and angle of force inclination were calculated as shown in Figure 2 and were averaged over the ground contact phase (N. Bezodis et al. 2021; Morin et al. 2011).

Kinematic variables were extracted from markerless motion capture using Visual3D. Shank angles were calculated from the proximal and distal endpoints of the tibia relative to the vertical axis (see Figure 2), where 0° represented a vertical shank and increasingly negative values represented greater negative shank inclination. COM height at touchdown was normalised to standing height. Sagittal COM to centre of pressure (COM‐COP) distance was normalised to leg length. Peak hip and knee sagittal plane angles were extracted during stance, with larger values representing greater joint flexion. Kinematic variables were selected based on previous conceptual frameworks and empirical evidence identifying postural adjustments as key determinants of horizontal force production and orientation during deceleration and change‐of‐direction tasks (Dos' Santos et al. 2020; Harper et al. 2022). HDA was quantified as the average rate of velocity reduction (i.e., deceleration) within each step, from touchdown to toe‐off, and cumulatively across early deceleration steps, from the first touchdown to the third toe‐off (early HDA).

### 2.5 Statistical Analysis

Descriptive data are presented as mean ± standard deviation (SD). For each participant, outcome variables were first averaged across all valid trials within each condition (i.e., speed and step), and then group‐level means and SDs were derived from these participant averages. Normality was assessed using the Shapiro‐Wilk test. Step (first, second, third) and speed (100%, 85% and 70%) differences were evaluated using a two‐way repeated measures ANOVA with Bonferroni‐corrected paired *t*‐tests for post‐hoc comparisons when significant main or interaction effects were found. Effect sizes were expressed as partial eta squared (*η*_*p*_^2^) and interpreted as small (> 0.01), medium (> 0.06) and large (> 0.14; Cohen 1988); statistical significance was set at *α* < 0.05.

Relationships between within‐step deceleration and kinetic variables were examined using Pearson's or Spearman's correlation coefficients, depending on data normality. Correlation strength was interpreted as: ≤ 0.1 trivial, > 0.1–0.3 small, > 0.3–0.5 moderate, > 0.5–0.7 large, > 0.7–0.9 very large, and > 0.9 almost perfect (Hopkins 2002). Mean horizontal GRF (HGRF) and mean H‐V force ratio were then analysed against whole‐body and joint kinematics using the same correlation criteria. Given the number of correlation analyses (*n* = 126), the Benjamini‐Hochberg (BH) method was used to control the false discovery rate (*q* = 0.05); correlations with BH‐adjusted *p* < 0.05 were considered significant (Benjamini and Hochberg 1995).

Multiple linear regression was used to assess the contribution of each deceleration step to early deceleration ability. Separate models were created for within‐step deceleration, mean HGRF and mean H‐V force ratio, with step‐specific values entered simultaneously. All variables were standardised to *z* scores. Multicollinearity was assessed using variance inflation factors (VIF < 5; James et al. 2013), model fit was evaluated using adjusted *R* ^2^, and statistical significance was set at *α* < 0.05.

All ANOVA and post‐hoc analyses were conducted in SPSS (v.28.0), and all correlation and regression analyses were performed using Python (v.3.12).

## 3 Results

Of the 285 trials obtained across 19 participants, 256 were retained for analysis. Several trials were excluded due to incorrect approach speed, an insufficient number (i.e., < 3) of valid deceleration steps, or first ground contact with non‐dominant limb. Horizontal approach velocities were 7.09 ± 1.1 m/s (100%), 6.02 ± 1.0 m/s (85%), and 5.09 ± 0.85 m/s (70%). Early deceleration was −5.07 ± 0.79 m/s^2^ (100%), −5.07 ± 1.12 m/s^2^ (85%), and −4.25 ± 0.79 m/s^2^ (70%).

**FIGURE 3:**
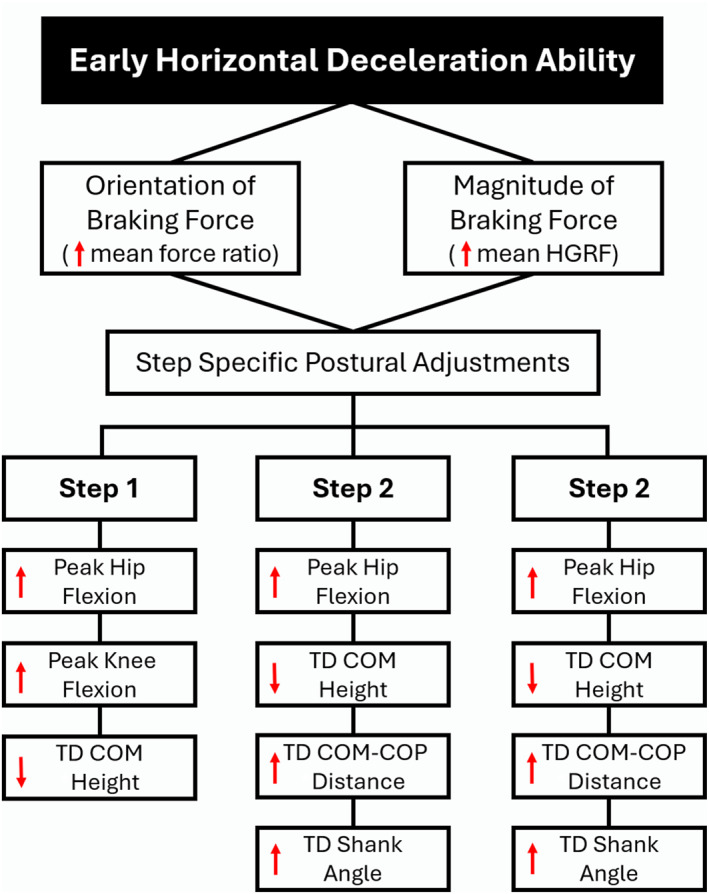
Step‐specific biomechanical determinants of early HDA. Schematic adapted from Harper et al. (2022), with adjustments to emphasise key kinetic predictors (mean HGRF and mean H‐V force ratio) and to incorporate step‐specific postural adjustments. Read bottom‐to‐top: postural adjustments at each step influence horizontal force magnitude and orientation, which in turn determine early HDA. Symbols: ↑ = increase; ↓ = decrease; COM = centre of mass; COP = centre of pressure; TD = touchdown.

Correlation analyses revealed that greater mean HGRF and H‐V force ratio were associated with greater within‐step deceleration across all speeds and steps (*r* = 0.56–0.95, *p* = 0.001–0.013; Figure 3: Supporting Information S1: Table A1). Correlations for both variables were generally stronger at slower approach speeds and later steps (i.e., steps 2 and 3). Horizontal impulse and peak HGRF showed inconsistent and weaker correlations to within‐step deceleration compared to mean HGRF and mean H‐V force ratio.

The regression analysis revealed that all three within‐step deceleration measures contributed to early deceleration ability (*R* ^2^ = 0.89–0.95). Mean HGRF significantly predicted early deceleration in step 1 across all speeds (*β* = 0.58–0.83, *p* < 0.05), whereas mean H–V force ratio was significant in both steps 1 and 3, with stronger effects in step 3 (*β* = 0.50–0.68, *p* < 0.05). No kinetic variable from step 2 was predictive of early deceleration ability at any speed. See Table A2 in the Supporting Information S1 for full regression models.

Further correlation analysis between whole‐body and joint kinematics to external kinetics revealed that greater peak hip and knee flexion and a lower COM height was significantly associated with greater mean HGRF and mean H‐V force ratio in step 1 (*r* = 0.57–0.80, *p* = 0.001–0.011). In steps 2 and 3, greater COM‐COP distance and peak hip flexion, a more negative shank inclination and a lower COM height were significantly associated with higher H‐V force ratios and mean HGRF (*r* = 0.49–0.9, *p* = 0.001–0.033). Correlations were generally weaker and often non‐significant at 100% approach speed. The complete correlation matrices corresponding to the key relationships are presented in Supporting Information S1: Tables A3 and A4 and visualised in Figure 3.

**FIGURE 4:**
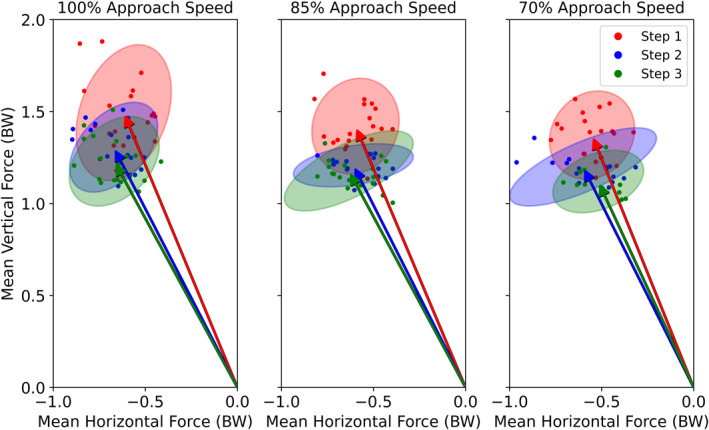
Graphical depiction of mean GRF vector at step 1 (black), step 2 (red) and step 3 (blue) at 100% (left), 85% (middle), 70% (right) approach speeds. Small dots depict individual participants' (*n* = 19) responses (averaged over multiple trials). Thick coloured arrows depict the mean force vectors. The ellipses represent within‐group covariance, with the radii scaled to encompass approximately 95% of the data distribution. Each ellipse is centred on the mean force vector and aligned with the direction of greatest variance. The relative magnitudes displayed on the vertical and horizontal axes were kept constant to aid visual comparison. Vector analysis and visualisation inspired by Kadlec et al. (2024).

**FIGURE 5:**
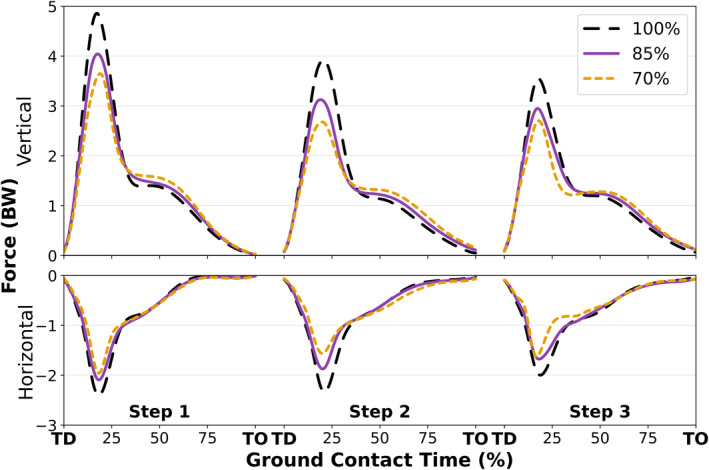
Vertical (top row) and horizontal (bottom row) ground reaction force (GRF) profiles during horizontal decelerations performed from 100% (black), 85% (purple), and 70% (yellow) approach speeds. Data are presented across the first (left), second (middle), and third (right) deceleration steps. Force traces represent the average of all participants' trials (*n* = 19), time‐normalised to 100% of stance duration.

Repeated measures ANOVA and post‐hoc analysis revealed distinct step effects; full results are presented in Supporting Information S1: Table A5. Deceleration increased in step 2 and stayed elevated in step 3 (*η*_*p*_^2^ = 0.18, *p* = 0.028). Change in COM velocity increased with each step (*η*_*p*_^2^ = 0.39, *p* = 0.001). Mean HGRF showed no step effect (*η*_*p*_^2^ = 0.05, *p* = 0.405). Mean H‐V force ratio and force inclination angle were greater in steps 2 and 3 compared to step 1 across all speeds (*η*_*p*_^2^ = 0.52, *p* = 0.001; Figure 4). Peak vertical GRF decreased across steps, while peak HGRF remained elevated in steps 1 and 2 then decreased at step 3 (*η*_*p*_^2^ = 0.21, *p* = 0.014; Figure 5). Horizontal impulse increased with step (*η*_*p*_^2^ = 0.20, *p* = 0.02). COM height decreased with step (*η*_*p*_^2^ = 0.71, *p* = 0.001). Shank angle showed no step effect (*η*_*p*_^2^ = 0.12, *p* = 0.098). Peak hip flexion increased with step (*η*_*p*_^2^ = 0.22, *p* = 0.017), whereas peak knee flexion had no significant differences (*η*_*p*_^2^ = 0.14, *p* = 0.09).

Distinct speed differences were evident across most variables. Change in COM velocity increased when approach speed decreased (*η*_*p*_^2^ = 0.46, *p* = 0.001). Mean HGRF remained elevated at 100% and 85% before reducing at 70% (*η*_*p*_^2^ = 0.55, *p* = 0.001; Figure 4). Both peak vertical and horizontal GRF increased with speed (*η*_*p*_^2^ = 0.80, *p* = 0.001; Figure 5). Horizontal impulse decreased with speed (*η*_*p*_^2^ = 0.30, *p* = 0.002). COM height was highest at 70% (*η*_*p*_^2^ = 0.24, *p* = 0.007). COM‐COP distance was greater at 100% and 85% compared to 70% (*η*_*p*_^2^ = 0.42, *p* = 0.001). Peak hip and knee flexion increased with speed (*η*_*p*_^2^ = 0.28, *p* = 0.003 and *η*_*p*_^2^ = 0.54, *p* = 0.001, respectively).

## 4 Discussion

The aim of this study was to (1) identify the step‐ and speed‐specific external kinetic determinants and underpinning kinematic strategies of HDA, and (2) compare kinetic and kinematic characteristics across early deceleration steps and approach speeds. The findings supported the hypothesis that the relationships between kinematic strategies and the kinetic determinants underpinning HDA became stronger during later deceleration steps and at slower approach speeds, reflecting a progressive reduction in whole‐body momentum and braking demands. A key and novel finding of this study was the identification of distinct step‐specific kinematic strategies underpinning horizontal force production. Specifically, step 1 required greater peak hip and knee flexion, whereas steps 2 and 3 relied more on optimally aligned touchdown postures (e.g., lower COM, greater COM–COP distance, and a more negative shank inclination) to enhance the magnitude and orientation of horizontal force.

Greater mean HGRF and mean H‐V force ratio consistently emerged as the strongest predictors of within‐step deceleration across all approach speeds and steps (except step 2 at 100%). This finding is not surprising as it is consistent with Newton's second law: greater horizontal force facilitates larger changes in (negative) acceleration. Our results also extend prior work on the kinetic determinants of HDA (Li et al. 2025), demonstrating that these kinetic determinants (i.e., mean HGRF and H‐V force ratio) are evident across a range of approach speeds and early deceleration steps. Notably, kinetic contributions to early HDA were found to be step‐specific: mean HGRF was the dominant predictor in step 1, whereas the mean H‐V force ratio contributed most during steps 1 and 3. These results suggest that when the whole‐body momentum is the greatest (i.e., step 1), producing and sustaining high horizontal forces is especially critical, whereas in later steps, as momentum decreases, effective horizontal force orientation becomes increasingly important. Given that the production of horizontal force is closely influenced by technical execution (Morin et al. 2011), coaching strategies (i.e., movement retraining) for multidirectional team sport athletes should reflect the distinct mechanical demands of each deceleration step.

In the first deceleration step, greater peak hip and knee flexion angles were significantly correlated with greater mean HGRF and mean H‐V force ratio across all approach speeds. These findings suggest that adopting a more flexed lower‐limb posture in late stance may enhance an athlete's ability to orient and sustain high horizontal forces. While previous work in change of direction (Dos' Santos et al. 2020), gait termination (Iqbal and Pai 2000), and horizontal deceleration (Harper et al. 2022) has similarly highlighted the role of joint flexion in braking mechanics, the present study extends these insights by showing that peak joint flexion was more strongly related to horizontal force production than touchdown kinematics in step 1. It is likely that greater hip and knee flexion in late stance facilitates a lower COM, prolongs ground contact time, and maintains a more favourable orientation of the GRF vector throughout the whole step (Dos' Santos et al. 2020). Accordingly, coaching practitioners are recommended to promote greater hip and knee flexion during the initiating deceleration step, as this posture appears critical for orientating and sustaining horizontal forces.

In the second and third deceleration steps, touchdown kinematics emerged as strong correlates to mean HGRF and mean H‐V force ratio across all speeds. Specifically, a lower COM height, greater COM‐COP distance (i.e., increased anterior foot placement) and a more negative shank inclination at touchdown were associated with higher mean HGRF and mean H‐V force ratios, with stronger associations observed at slower speeds. While the touchdown postures have previously been identified as important across the whole deceleration sequence (Harper et al. 2022), the present findings are the first to suggest that touchdown kinematics are particularly critical during later deceleration steps and at slower approach speeds—both of which are characterised by a lower COM velocity. The stronger associations when momentum is reduced likely reflect the athlete's increased ability to adopt mechanically advantageous postures due to lower braking demands. During the initial braking steps, athletes are required to rapidly transition from sprinting to decelerating while simultaneously attenuating a large momentum. This limits their ability to consistently achieve optimal touchdown positions. As momentum progressively decreases, however, athletes may be better able to orient the lower limb relative to the COM, thereby facilitating more effective horizontal force orientation. Based on these findings, practitioners are recommended to assess and coach key touchdown characteristics (e.g., lower COM position, greater COM‐COP distance, and a more negative shank inclination) in later deceleration steps. This is especially important during high‐speed deceleration drills, where rapidly transitioning into such postures becomes more technically challenging.

While previous studies have explored kinematic responses to horizontal deceleration across varying approach speeds (Chen et al. 2024; Jordan et al. 2021; Norman et al. 2025; Philipp et al. 2023), the present study is among the first to examine kinetic changes. Notably, mean HGRF did not significantly change across steps despite progressive reductions in whole‐body momentum. This initially counterintuitive finding may be explained by changes in force orientation: both the mean H‐V force ratio and angle of force inclination were lowest in step 1 and increased in steps 2 and 3, enabling athletes to maintain high horizontal force despite declining momentum. Consequently, larger reductions in COM velocity (10%–30%) and greater deceleration (6%–14%) were observed in steps 2 and 3 compared with step 1. Step 1 may, therefore, function as a preparatory step as athletes transition out of high‐speed running, during which deceleration ability is constrained by minimal adjustments to touchdown postures (Harper et al. 2022; Havens and Sigward 2015). This interpretation is reinforced by the weak and often non‐significant correlations between touchdown kinematics and horizontal force orientation in step 1, suggesting that the transitional nature of this step makes it technically challenging for athletes to adopt touchdown postures that contribute meaningfully to horizontal force production. Such a preparatory step may increase braking demands in later steps, prolong stopping time, and ultimately limit HDA (Harper et al. 2021). This raises an important question for practitioners: can horizontal force production and orientation be enhanced in the first deceleration step, or is it an unavoidable consequence of exiting high‐speed running? While this study cannot directly answer this question, future research could investigate whether movement‐retraining interventions targeting technical execution at touchdown, or unanticipated deceleration drills that challenge decision‐making and coordination (Harper et al. 2024), can improve deceleration postures and force orientation during the first deceleration step, at which running speed is the highest.

High‐intensity horizontal decelerations are recognised as some of the most mechanically demanding locomotor tasks in terms of external force characteristics (Lozano‐Berges et al. 2021). The present findings extend this understanding by demonstrating that even decelerations performed from submaximal approach speeds (70% and 85%) impose substantial external loads. For instance, peak vertical GRFs at 70% and 85% (4.1 and 4.6 times BW, respectively) were lower than at maximal speeds (5.3 BW) but remained comparable to values observed during high‐speed running (4.4 BW) and almost double those typical during acceleration (2.2 BW) (I. N. Bezodis et al. 2008; Verheul et al. 2021). Additionally, vertical and horizontal impulses increased as approach speed decreased, likely reflecting longer ground contact times when momentum reduces (Dos’ Santos et al. 2021). Collectively, these findings indicate that submaximal decelerations still present a considerable mechanical challenge and may contribute to muscle damage and neuromuscular fatigue that impair coordination and deceleration ability, particularly in the latter stages of match play (Edwards 2018; McBurnie et al. 2022). Accordingly, coaching practitioners are recommended to incorporate deceleration drills across a range of approach speeds to build athletes' tolerance to both high‐speed and submaximal external braking demands.

Some caution should be exercised in the interpretation and generalisation of the key findings of this study. First, the deceleration task used in the present study was pre‐planned, allowing participants sufficient time to prepare for the braking manoeuvre and potentially adopt appropriate postural adjustments prior to the initiation of deceleration. Consequently, the findings may not fully reflect the biomechanical demands of reactive or unplanned decelerations commonly observed during multidirectional team sports. Future studies should examine whether the kinetic and kinematic determinants identified in the present study are maintained under more ecologically valid, perceptually demanding conditions, thereby informing more game‐specific training interventions. Second, while the whole deceleration sequence typically involves up to six braking steps, this study focused on early deceleration ability (i.e., first three steps). Future investigations capturing all braking contacts would offer a more comprehensive understanding of the step‐specific contributions to overall HDA. Third, validations of Theia3D during high‐intensity deceleration tasks remain limited. Consequently, comparisons of absolute kinematic values with studies using different motion‐capture systems should be made cautiously. While marker‐based motion capture is commonly regarded as the gold standard, no true ground‐truth measure exists for high‐intensity decelerations, particularly given that the rapid limb accelerations and increased soft tissue artefact associated with these movements may limit the accuracy of marker‐based motion capture. Finally, this study focused exclusively on external kinetics and kinematics, providing insight into technical ability but not the underlying internal loading mechanisms. Assessing joint‐level mechanics and tissue‐level forces (Verheul et al. 2024) across multiple steps and approach speeds will further deepen our understanding of internal braking demands and inform targeted training strategies that enhance eccentric strength, inter‐limb coordination, and neuromuscular control during horizontal decelerations.

## 5 Conclusions

This study provided the first unified step‐ and speed‐specific analysis of the external kinetic and kinematic determinants of early HDA, identifying how mechanical demands and optimal braking strategies shift across steps and approach speeds. Practitioners should aim to maximise horizontal force magnitude (i.e., mean HGRF) and horizontal force orientation (i.e., mean H‐V force ratio), particularly in steps 1 and 3, respectively, to enhance early HDA. To optimise these kinetic variables, practitioners are recommended to coach greater hip and knee flexion during the first deceleration step and promote technically optimal touchdown postures in later steps, such as a lower COM, increased anterior foot placement, and a more negative shank inclination. Finally, this study demonstrated that submaximal horizontal decelerations (70% and 85% approach speeds) still impose substantial mechanical demands, characterised by high braking forces and impulses. Practitioners should therefore prepare athletes for these demands by incorporating deceleration drills across a range of approach speeds.

## Funding

The authors have nothing to report.

## Ethics Statement

This study was approved by Cardiff Metropolitan Natural Ethics Committee (Code: PGT‐9450) and all study procedures were conducted according to the Declaration of Helsinki.

## Consent

All participants provided written informed consent prior to participation.

## Conflicts of Interest

The authors declare no conflicts of interest.

## Permission to Reproduce Material From Other Sources

The authors have nothing to report.

## Supporting information


Supporting Information S1

## Data Availability

The data that support the findings of this study are available from the corresponding author, upon reasonable request.
