# Supplementary material for: Early Horizontal Deceleration Ability Across Multiple Steps and Approach Speeds in Multidirectional Team Sport Athletes
Source: Eur J Sport Sci. 2026 Jul 28;26(8):e70224. doi: 10.1002/ejsc.70224 (PMC13408429; doi:10.1002/ejsc.70224)
Supplement: Supplementary file 1 — Supporting Information S1 [file EJSC-26-e70224-s001.docx]

**Appendix**

**Table A1**: Correlations between within-step deceleration (m/s²) and GRF variables across three approach speeds (100%, 85%, 70%) and three deceleration steps.

| Speed | Step | Peak HGRF | Mean HGRF | H Impulse | Mean H-V Ratio |  |
| --- | --- | --- | --- | --- | --- | --- |
|  |  |  |  |  |  |  |
| 100 | 1 | 0.3 (M) | **0.62 (L)**** | 0.41 (M) | **0.79 (VL)**** |  |
|  | 2 | 0.11 (S) | **0.51 (L)**** | 0.05 (T) | 0.49 (M)* |  |
|  | 3 | -0.05 (T) | **0.56 (L)**** | 0.28 (S) | **0.71 (VL)**** |  |
|  |  |  |  |  |  |  |
| 85 | 1 | 0.23 (S) | **0.86 (VL)**** | 0.48 (M)* | **0.68 (L)**** |  |
|  | 2 | 0.4 (M) | **0.73 (VL)**** | **0.53 (L)**** | **0.84 (VL)**** |  |
|  | 3 | 0.44 (M) | **0.92 (AP)**** | **0.66 (L)**** | **0.84 (VL)**** |  |
|  |  |  |  |  |  |  |
| 70 | 1 | 0.39 (M) | **0.88 (VL)**** | **0.52 (L)**** | **0.72 (VL)**** |  |
|  | 2 | 0.38 (M) | **0.87 (VL)**** | **0.69 (L)**** | **0.81 (VL)**** |  |
|  | 3 | 0.45 (M) | **0.83 (VL)**** | 0.36 (M) | **0.95 (AP)**** |  |

Values represent r (magnitude descriptor). * Indicates *p*<0.05 (uncorrected); ** indicates significant after Benjamini-Hochberg (BH) false discover rate (q=0.05). Cells are shaded for B-H corrected significant correlations of moderate (orange), large (light green), very large (green), and almost perfect (dark green) magnitude. HGRF = horizontal ground reaction force, H = horizontal, H-V = horizontal to vertical, T = trivial, S = small, M = moderate, L = large, VL = very large, AP = almost perfect.

**Table A2**: Results of the multiple linear regression analyses with early deceleration as the dependent variable, and within-step deceleration, mean HGRF and mean H-V force ratio as independent variables, across three approach speeds (100%, 85%, 70%) and three deceleration steps.

| Variable | Speed | ADJ. R² | Step 1 | Step 2 | Step 3 |
| --- | --- | --- | --- | --- | --- |
| Within-step DEC | 100 | 0.89 | **0.40**** | **0.20**** | **0.29**** |
|  | 85 | 0.95 | **0.26**** | **0.34**** | **0.59**** |
|  | 70 | 0.87 | **0.35**** | 0.23** | **0.34**** |
|  |  |  |  |  |  |
| Mean HGRF | 100 | 0.61 | **0.80**** | 0.02** | 0.01** |
|  | 85 | 0.86 | **0.58**** | 0.08** | **0.42**** |
|  | 70 | 0.87 | **0.83**** | 0.13** | 0.15** |
|  |  |  |  |  |  |
| Mean H-V Ratio | 100 | 0.79 | 0.19** | 0.25** | **0.61**** |
|  | 85 | 0.79 | **0.41**** | 0.17** | **0.50**** |
|  | 70 | 0.86 | **0.48**** | 0.03** | **0.68**** |

Values represent standardised beta coefficients, * indicates *p*<0.05, ** indicates *p*<0.01. DEC = deceleration, HGRF = horizontal ground reaction force, H-V = horizontal-to-vertical.

**Table A3**: Correlations between mean HGRF and whole-body/joint kinematics across three approach speeds (100%, 85%, 70%) and three deceleration steps.

| Speed | Step | Shank Angle | COM-COP Distance | COM Height | Peak Hip Flexion | Peak Knee Flexion |  |
| --- | --- | --- | --- | --- | --- | --- | --- |
|  |  |  |  |  |  |  |  |
| 100 | 1 | 0.45 (M) | 0.49 (M)* | -0.39 (M) | **0.68 (L)**** | **0.66 (L)**** |  |
|  | 2 | 0.25 (S) | 0.11 (S) | -0.26 (S) | 0.39 (M) | 0.35 (M) |  |
|  | 3 | **0.58 (L)**** | **0.57 (L)**** | -0.15 (S) | 0.37 (M) | 0.1 (T) |  |
|  |  |  |  |  |  |  |  |
| 85 | 1 | **0.62 (L)**** | **0.61 (L)**** | -0.36 (M) | **0.69 (L)**** | **0.74 (VL)**** |  |
|  | 2 | 0.46 (M) | 0.39 (M) | -0.43 (M) | **0.59 (L)**** | 0.27 (S) |  |
|  | 3 | **0.84 (VL)**** | **0.7 (VL)**** | **-0.58 (L)**** | **0.72 (VL)**** | 0.22 (S) |  |
|  |  |  |  |  |  |  |  |
| 70 | 1 | **0.6 (L)**** | 0.5 (L)* | -0.41 (M) | **0.67 (L)**** | **0.69 (L)**** |  |
|  | 2 | **0.67 (L)**** | **0.6 (L)**** | -0.53 (L)* | **0.72 (VL)**** | 0.07 (T) |  |
|  | 3 | **0.85 (VL)**** | **0.87 (VL)**** | -0.26 (S) | 0.60 (L)* | 0.5 (L) |  |

Values represent *r* (magnitude descriptor). * indicates *p*<0.05 (uncorrected); ** indicates significant after Benjamini-Hochberg (BH) false discover rate (q=0.05). Cells are shaded for B-H corrected significant correlations of large (light green), very large (green), and almost perfect (dark green) magnitude. *COM = centre of mass, COP = centre of pressure, T = trivial, S = small, M = moderate, L = large, VL = very large, AP = almost perfect.*

**Table A4**: Correlations between mean H-V force ratio and whole-body/joint kinematics across three approach speeds (100%, 85%, 70%) and three deceleration steps.

| Speed | Step | Shank Angle | COM-COP Distance | COM Height | Peak Hip Flexion | Peak Knee Flexion |  |
| --- | --- | --- | --- | --- | --- | --- | --- |
|  |  |  |  |  |  |  |  |
| 100 | 1 | 0.28 (S) | 0.33 (M) | **-0.61 (L)**** | **0.59 (L)**** | **0.63 (L)**** |  |
|  | 2 | 0.16 (S) | 0.28 (S) | -0.48 (M)* | **0.59 (L)**** | **0.54 (L)**** |  |
|  | 3 | **0.49 (M)**** | **0.61 (L)**** | **-0.64 (L)**** | **0.66 (L)**** | 0.32 (M) |  |
|  |  |  |  |  |  |  |  |
| 85 | 1 | 0.45 (M) | 0.36 (M) | **-0.57 (L)**** | **0.64 (L)**** | **0.68 (L)**** |  |
|  | 2 | **0.51 (L)**** | **0.52 (L)**** | **-0.54 (L)**** | **0.68 (L)**** | 0.44 (M) |  |
|  | 3 | **0.76 (VL)**** | **0.78 (VL)**** | **-0.71 (VL)**** | **0.83 (VL)**** | 0.48 (M)* |  |
|  |  |  |  |  |  |  |  |
| 70 | 1 | 0.36 (M) | 0.2 (S) | **-0.63 (L)**** | **0.8 (VL)**** | **0.66 (L)**** |  |
|  | 2 | **0.56 (L)**** | **0.62 (L)**** | **-0.57 (L)**** | **0.78 (VL)**** | 0.34 (M) |  |
|  | 3 | 0.6 (L) | **0.9 (AP)**** | **-0.58 (L)**** | **0.72 (VL)**** | **0.66 (L)**** |  |

Values represent *r* (magnitude descriptor). * indicates *p*<0.05 (uncorrected); ** indicates significant after Benjamini-Hochberg (BH) false discover rate (q=0.05). Cells are shaded for B-H corrected significant correlations of moderate (orange), large (light green), very large (green), and almost perfect (dark green) magnitude. *COM = centre of mass, COP = centre of pressure, trivial, S = small, M = moderate, L = large, VL = very large, AP = almost perfect.*

**Table A5**: Descriptive statistics and ANOVA results for performance, kinetic and kinematic variables across the three approach speeds and deceleration steps. For ANOVA results, data is presented as effect size (p value), with significant effects/interaction highlighted in bold.

|  |  | Mean (SD) | | | Main Effect | | Interaction |
| --- | --- | --- | --- | --- | --- | --- | --- |
|  | Speed | Step 1 | Step 2 | Step 3 | Speed | Step | Speed×Step |
| Performance Variables | | | | | | | |
| Within-Step DEC (m/s²) | 100 | -4.99 (1.53) | -5.71 (1.10) | -5.56 (0.88) | **0.19 (0.025)** | **0.18 (0.028)** | 0.12 (0.052) |
|  | 85 | -5.27 (1.64) | -5.56 (1.23) | -5.53 (1.67) |  |  |  |
|  | 70 | -4.91 (1.40) | -5.55 (1.57) | -4.55 (0.95) |  |  |  |
|  |  |  |  |  |  |  |  |
| ∆COM Velocity (m/s) | 100 | -0.68 (0.22) | -0.81 (0.21) | -0.89 (0.31) | **0.46 (<0.001)** | **0.39 (<0.001)** | 0.11 (0.089) |
|  | 85 | -0.82 (0.25) | -0.95 (0.32) | -1.07 (0.32) |  |  |  |
|  | 70 | -0.89 (0.30) | -1.06 (0.35) | -0.98 (0.20) |  |  |  |
| Kinetic Variables | | | | | | | |
| Peak HGRF (BW) | 100 | -2.97 (0.65) | -2.97 (0.74) | -2.72 (0.80) | **0.80 (<0.001)** | **0.21 (0.014)** | **0.16 (0.013)** |
|  | 85 | -2.69 (0.48) | -2.40 (0.70) | -2.29 (0.79) |  |  |  |
|  | 70 | -2.37 (0.57) | -1.97 (0.70) | -1.76 (0.71) |  |  |  |
|  |  |  |  |  |  |  |  |
| Mean HGRF (BW) | 100 | -0.62 (0.13) | -0.67 (0.12) | -0.67 (0.12) | **0.55 (<0.001)** | 0.05 (0.405) | 0.15 (0.05) |
|  | 85 | -0.60 (0.12) | -0.61 (0.16) | -0.63 (0.17) |  |  |  |
|  | 70 | -0.55 (0.12) | -0.60 (0.20) | -0.52 (0.12) |  |  |  |
|  |  |  |  |  |  |  |  |
| H Impulse (BW·s) | 100 | -0.09 (0.02) | -0.10 (0.02) | -0.11 (0.04) | **0.30 (0.002)** | **0.20 (0.02)** | **0.20 (0.028)** |
|  | 85 | -0.10 (0.02) | -0.11 (0.03) | -0.13 (0.03) |  |  |  |
|  | 70 | -0.11 (0.03) | -0.12 (0.04) | -0.12 (0.03) |  |  |  |
|  |  |  |  |  |  |  |  |
| Mean H-V Force Ratio (%) | 100 | -32.7 (10.2) | -43.1 (7.4) | -45.6 (8.3) | 0.14 (0.07) | **0.52 (<0.001)** | **0.23 (0.003)** |
|  | 85 | -33.9 (8.8) | -41.3 (9.4) | -44.3 (7.5) |  |  |  |
|  | 70 | -33.7 (9.2) | -41.0 (9.9) | -39.1 (7.8) |  |  |  |
|  |  |  |  |  |  |  |  |
| Inclination Angle (°) | 100 | -19.2 (6.3) | -25.7 (4.7) | -27.3 (5.3 | 0.14 (0.06) | **0.52 (<0.001)** | **0.24 (<0.001)** |
|  | 85 | -20.0 (5.4) | -24.6 (6.0) | -26.4 (4.8) |  |  |  |
|  | 70 | -19.8 (5.6) | -24.4 (6.3) | -23.1 (4.9) |  |  |  |
| Kinematic Variables | | | | | | | |
| Shank Angle (°) | 100 | -23.7 (4.3) | -26.2 (4.7) | -26.4 (4.1) | **0.24 (0.007)** | 0.12 (0.098) | **0.25 (0.006)** |
|  | 85 | -25.5 (4.4) | -26.7 (5.9) | -27.6 (5.4) |  |  |  |
|  | 70 | -24.7 (3.6) | -25.7 (6.5) | -23.2 (4.1) |  |  |  |
|  |  |  |  |  |  |  |  |
| COM-COP Distance (%LL) | 100 | 0.50 (0.07) | 0.51 (0.08) | 0.51 (0.05) | **0.42 (<0.001)** | 0.02 (0.758) | 0.06 (0.391) |
|  | 85 | 0.51 (0.08) | 0.50 (0.09) | 0.50 (0.09) |  |  |  |
|  | 70 | 0.49 (0.07) | 0.48 (0.09) | 0.46 (0.06) |  |  |  |
|  |  |  |  |  |  |  |  |
| COM Height (%SH) | 100 | 0.50 (0.01) | 0.48 (0.02) | 0.47 (0.02) | **0.24 (0.007)** | **0.71 (<0.001)** | 0.06 (0.348) |
|  | 85 | 0.50 (0.02) | 0.48 (0.02) | 0.47 (0.02) |  |  |  |
|  | 70 | 0.50 (0.02) | 0.49 (0.02) | 0.48 (0.03) |  |  |  |
|  |  |  |  |  |  |  |  |
| Peak Hip Flexion (°) | 100 | 42.5 (6.7) | 45.0 (6.9) | 47.5 (8.2) | **0.28 (0.003)** | **0.22 (0.017)** | **0.25 (0.004)** |
|  | 85 | 41.4 (5.0) | 43.3 (7.1) | 45.1 (7.4) |  |  |  |
|  | 70 | 40.6 (5.3) | 42.2 (7.1) | 40.4 (8.1) |  |  |  |
|  |  |  |  |  |  |  |  |
| Peak Knee Flexion (°) | 100 | 87.8 (8.0) | 91.5 (8.2) | 88.9 (14.9) | **0.54 (<0.001)** | 0.14 (0.09) | 0.11 (0.127) |
|  | 85 | 87.4 (8.8) | 89.0 (12.6) | 83.2 (17.9) |  |  |  |
|  | 70 | 85.0 (10.3) | 80.9 (15.9) | 78.5 (12.3) |  |  |  |

SD = standard deviation, DEC = deceleration, COM = centre of mass, HGRF = horizontal ground reaction force, BW = body weight, H = horizontal, H-V = horizontal-to-vertical, COP = centre of pressure, %LL = percentage of leg length, %SH = percentage of standing height.
